# Proteomic Profiling of Acoustically Isolated Extracellular Vesicles from Blood Plasma during Murine Bacterial Sepsis

**DOI:** 10.1021/acs.jproteome.5c00267

**Published:** 2025-07-11

**Authors:** Axel Broman, Yashuan Chao, Oonagh Shannon, Thomas Laurell, Johan Malmström

**Affiliations:** † Department of Biomedical Engineering, Lund University, Lund 222 42, Sweden; ‡ Division of Infection Medicine, Department of Clinical Sciences, Lund University, Lund 222 42, Sweden; § Section of Oral Biology, Faculty of Odontology, 59607 Malmö University, Malmö 205 06, Sweden

**Keywords:** extracellular vesicles, sepsis, acoustic trapping, proteomics, mass spectrometry

## Abstract

Sepsis is a life-threatening condition caused by a dysregulated host response to an infection and is a leading cause of death worldwide. The condition is variable, which in combination with insufficient clinical markers, makes it challenging to predict when infection will progress to sepsis and to categorize patients into homogeneous patient subgroups. In this study, we demonstrate the use of acoustic trapping to rapidly enrich extracellular vesicles (EVs) from minute volumes of blood plasma from experimental mouse models of sepsis infected with the Gram-positive pathogen or the Gram-negative pathogen . Using quantitative mass spectrometry-based proteomics, we characterized the proteome of EVs and plasma to demonstrate that the EVs expand the observable proteome in plasma, with an emphasis on cellular processes and signaling. In our models, systemic bacterial infection altered the EV and plasma proteomes differently, with a predominant effect on proteins related to leukocyte migration in the EVs and on metabolism in the plasma. Finally, we show that infection significantly impacted metabolism, whereas infection mainly affected the inflammatory response and neutrophil degranulation in our models. Collectively, our findings demonstrate that the acoustic trap facilitates access to plasma EVs, which in turn provides additional biological information which was not obtained from the plasma proteome alone.

## Graphical abstract

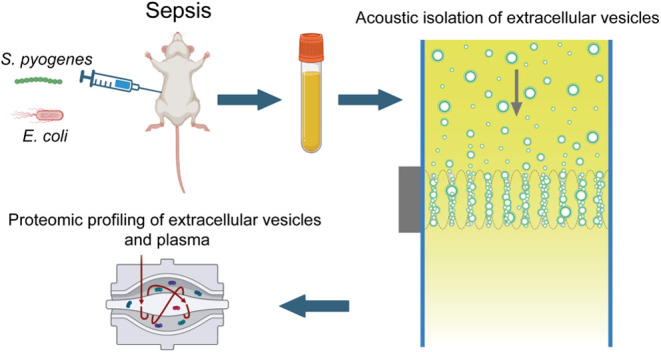

## Introduction

Sepsis is a life-threatening condition caused by a dysregulated host response to infection leading to organ dysfunction.^1^ The disease is currently a major cause of mortality and is estimated to contribute to 19% of deaths worldwide.^2^ As such, there is a need for better understanding and treatment of sepsis. However, in the previous four decades, over 120 treatment trials for sepsis have been unsuccessful.^3^ One reason for this is the heterogeneous nature of the syndrome that reflects the complex and systemic host response that depends on many factors, such as type of pathogen, site of infection, and time elapsed since infection.^4,5^ Bacterial pathogens such as and (Group A Streptococcus, GAS) can cause sepsis when systemic infection dysregulates the immune response. To advance the understanding of the pathophysiology in sepsis, there is a need to further characterize the biological processes involved in sepsis.

Studies in preclinical models of infections have shown that sepsis causes systems-wide proteome changes in several organs and cell systems.^3^ Typically, these proteome changes are variable and depend on time elapsed since infection, type of pathogen, and type of treatment.^3,6,7^ The variability, in combination with insufficient clinical markers to accurately predict when an infection will progress to sepsis or categorize patients into homogeneous patient subgroups, makes it challenging to perform clinical intervention trials.

Extracellular vesicles (EVs) are small membrane-encapsulated particles released by cells into the extracellular environment. EVs range in size from 30–1000 nm and carry a diverse biological cargo of RNAs, proteins, and metabolites that reflect their cell of origin.^8,9^ EVs can play a role as messengers, carrying and delivering their contents between cells, and play a role in many biological functions, such as cell–cell communication, immune response, homeostasis, and coagulation.^8,9^ Sepsis introduces marked changes in the EV population, which have been associated with inflammation, apoptosis, bacterial clearance, and organ damage.^10^ However, the effect of EVs during sepsis has been shown to be both pro- and anti-inflammatory as well as pro- and antiapoptotic. These findings indicate that the role of EVs can change throughout the course of sepsis and depends on patient heterogeneity, type of pathogen, and when during disease progression the EVs were harvested. Changes in the EV population may consequently contain so far unexplored disease-relevant molecular signals that require larger scale studies to uncover, interfaced with more comprehensive analytical techniques to analyze the EV population and its cargo. Intriguingly, EVs can be isolated from biobanked plasma samples, which generates new opportunities to extract molecular information that is obscured by the high dynamic range associated with the plasma proteome. Increasing the understanding of EV signaling would help to elucidate the biological processes occurring during sepsis and represents an untapped source of novel biomarkers. However, exploring changes in the EV population requires EV isolation techniques that are fast, simple, robust and can be interfaced to proteomics, transcriptomics and metabolomics techniques, to more exhaustively quantify the changes of the EV cargo directly from clinical samples.

The current gold standards for EV isolation are ultracentrifugation (UC) and size-exclusion chromatography (SEC). However, these techniques have a few drawbacks. UC has several hour-long processing times, and the large forces may compromise EV integrity and induce aggregation.^11^ SEC operates much faster (∼15 min) and retains EV integrity,^12^ however the columns used will be biased toward EVs of a certain size. Additionally, SEC columns are either single or 5-times use, and there is risk of clogging in low-molecular weight filters.^13^ There is therefore a need for EV isolation techniques that are faster, reusable, and do not alter the characteristics of EVs during the isolation process.

Acoustic trapping is a technique for isolation and enrichment of EVs. In acoustic trapping, an ultrasonic standing wave is generated inside a microfluidic channel. Particles that are denser and less compressible than the surrounding fluid will move toward the pressure nodes, where they can be retained against a flow,^14^ allowing for isolation, enrichment, and washing of the particles. Acoustic trapping has successfully been used to isolate EVs from different biological fluids, including urine,^15–18^ conditioned media,^15^ cerebrospinal fluid^19^ and blood plasma.^15,20–24^ Proteomic analysis of isolated EVs has shown that acoustic trapping and ultracentrifugation produce very similar results.^22,23^ Electron microscopy and nanoparticle tracking analysis (NTA) has previously shown that acoustically isolated EV populations display greater emphasis on smaller EVs than EVs isolated by ultracentrifugation.^15^ We have recently reported on a new type of acoustic trapping platform with improvements in throughput and capacity, which allows for processing mL sample volumes in minutes.^18^ The gentle forces involved in acoustic trapping, in combination with fast processing time, few manual steps, and the ability to also isolate EVs from small sample volumes, makes acoustic trapping an attractive method for EV isolation. The ability to rapidly isolate EVs from small volumes enables studies on EVs that are practically not feasible with other methods requiring larger sample volumes and longer processing times.

In this work, we study changes in the EV proteome compared to the plasma proteome that occur during sepsis, by isolating EVs from blood plasma from experimental mouse models of sepsis with bacterial pathogens, GAS and . The EVs were isolated from 80 μL of blood plasma using an acoustic trapping platform. Interfacing the acoustic trap to high-resolution quantitative mass spectrometry-based proteomics uncovered different disease- and pathogen- specific proteome signatures in the EV population and the in the blood plasma.

## Experimental Section

### Bacterial Strains

 AP1 (from the Collection of the World Health Organization Collaborating Center for Reference and Research on Streptococci, Prague, Czech Republic) was grown to an optical density of 0.4 at 620 nm (OD_620_ = 0.4) in Todd-Hewitt broth supplemented with 0.2% yeast extract (THY). O18:K1 ^25^ was grown to an OD_620_ = 0.25 in Luria–Bertani broth (LB). Bacteria were grown at 37 °C with 5% CO_2_ without shaking. Bacteria were washed and resuspended in sterile PBS to 1 × 10^8^ CFU/mL for AP1 and 5 × 10^4^ CFU/mL for O18:K1. These bacterial growth conditions are adapted from previously established murine sepsis models for^26^ and ,^3^ and are used to generate a reproducible infectious dose.

### Intraperitoneal Infection with Bacteria in Mice

All animal experiments were approved by the local ethical committee (ethical permit number 11542–2020).

Nine-week-old female C57BL/6J mice (Janvier, Le Genest-Saint-Isle, France) were infected with 100 μL (1 × 10^7^ CFU) of AP1 or 200 μL (1 × 10^4^ CFU) of O18:K1 by intraperitoneal injection. The infectious dose for each bacterium is based on previously established models of murine sepsis for the distinct pathogens.^3,26^ Control mice received sterile PBS. Animals were monitored regularly for body weight and symptoms of illness. Animals were sacrificed at a predetermined time point of 18 h postinfection (h.p.i.). Animals with weight loss >15% were euthanized before the predetermined time point and were excluded from the study. Blood was taken by cardiac puncture and collected in tubes containing sodium citrate (MiniCollect tube, Geiner Bio-One). Liver and spleen were collected for determination of bacterial load.

### Flow Cytometry

Citrated blood (20 μL) was diluted with 30 μL of a solution containing 29 μL of HEPES buffer (pH 7.4; 10 mM HEPES, 150 mM NaCl, 5 mM KCl, and 1 mM MgSO_4_) and 1 μL of mouse Fc-block (BD Pharmingen). The following antibodies were used in a panel to determine white blood cell (WBC) counts, the formation of platelet-neutrophil complexes (PNCs) and platelet-monocyte complexes (PMCs), and the activation status of neutrophils and monocytes: per sample, 2.5 μL of each APC-R700 Rat Anti-Mouse Ly-6G Ly-6C (BD Horizon, Catalog No. 565510, Clone RB6–8C5); FITC Rat Anti-Mouse CD41 (BD Pharmingen, Catalog No. 553848, Clone MWReg30); PE Rat Anti-Mouse Ly-6G (BD Pharmingen, Catalog No. 551461, Clone 1A8); and PerCP-Cy5.5 Rat Anti-CD11b (BD Pharmingen, Catalog No. 550993, Clone M1/70). The following antibody was used in a panel to determine platelet counts: per sample, 2.5 μL of APC Rat Anti-Mouse CD41 (Biolegend, Catalog No. 133914, Clone MWReg30). The samples were stained with antibodies and incubated for 15 min at room temperature in the dark. Samples were incubated with 1 mL of 1-step Fix/Lyse Solution (e-Bioscience) for 30 min at room temperature and centrifuged at 500*g* for 5 min. For the platelet panel samples, a wash step with 1 mL of PBS (500*g* for 5 min) was included to remove excess red blood cell debris. For all samples, one mL of supernatant was carefully removed and discarded and the remaining volume with cells was resuspended in 500 μL of PBS. Samples were analyzed using an Accuri C6 Plus flow cytometer and software (BD Biosciences). For the WBC panel, linear settings were applied, the FSC-H threshold was set at 80,000, and 10,000 events were acquired per sample in the leukocyte gate. For the platelet panel, logarithmic settings were applied, the FSC-H threshold was set at 45,000 and 30,000 events were acquired per sample in the CD41-positive platelet gate.^27^ Representative gating strategies and histogram plots for samples from a healthy, an AP1-infected, and an O18:K1-infected animal 18 h.p.i. are shown in Figure S3.

### Preparation of Plasma and Organs

Citrated blood was centrifuged in a two-step preparation: 500*g* for 10 min to obtain platelet-rich plasma, then 2500*g* for 10 min to obtain platelet-free plasma (referred to as plasma). Plasma was aliquoted, with one set immediately processed on the acoustic trapping platform for EV isolation and another set stored at −80 °C until further analysis (see below). Liver and spleen were homogenized (MagnaLyzer, Roche) in sterile PBS using sterile silica beads (1 mm diameter, Techtum). Bacterial load in organ homogenates was determined by serial diluting and plating onto blood agar plates. CFUs were counted following overnight incubation at 37 °C with 5% CO_2_ and are presented as CFU/g of tissue.

### Quantification of IL-6 and Organ Damage Markers

Plasma levels of IL-6 (DuoSet ELISA, DY406, Bio-Techne, R&D Systems), lactate dehydrogenase (LDH) activity [LDH Assay Kit (colorimetric), ab102526, Abcam], and alanine transaminase (ALT) activity [ALT Assay Kit (colorimetric), ab105134, Abcam] were measured according to manufacturer’s instructions, with the exception that 100 μL of ELISA Stop Solution (Invitrogen) was used to stop the reaction in the ELISA. Plasma samples from healthy animals were diluted 1:20 in Reagent Diluent for IL-6 analysis and 1:40 in Assay Buffer for LDH analysis. Plasma samples from infected animals were diluted 1:100 in respective buffers for both IL-6 and LDH analyses. All plasma samples were diluted 1:10 in Assay Buffer for ALT analysis.

### Statistical Analysis for Pathophysiological Assays

Statistical analysis was performed using GraphPad Prism 10 software. Comparisons were analyzed for statistical significance using Mann–Whitney test. Results were considered significant for comparisons where *P* < 0.05.

### Isolation of Extracellular Vesicles Using Acoustic Trapping

A schematic overview of sample processing on the acoustic trapping platform is shown in Figure 3A. 80 μL of plasma was diluted by adding 320 μL of PBS, yielding a concentration of 20% plasma. Extracellular vesicles were then isolated from the plasma by processing on an acoustic trapping platform, described previously.^18^ Briefly, an ultrasonic transducer generates a standing wave inside a glass capillary. Particles in the vicinity of the wave will move toward the pressure node, where they can be captured and retained against flow, allowing for isolation, enrichment and washing of particles in a fluid (Figure 3A). The trap was actuated with 12 V_pp_ (peak-to-peak) and loaded with 500 μL of a solution containing 0.048% (w/v in PBS) 12 μm polystyrene seed particles (Sigma-Aldrich) at a flow rate of 1000 μL/min, to establish a seed particle cluster. The cluster was then washed with 1 mL PBS at a flow rate of 1000 μL/min to remove excess seed particles. The seed particles retained in the standing wave serve as a trapping site for EVs. Sample was then processed in the trap at a flow rate of 500 μL/min to isolate EVs in the seed particle cluster. While retained in the trap, the EVs were washed with a continuous flow of 5 mL of PBS at 500 μL/min to remove background plasma proteins. The ultrasound was then turned off and the seed particle cluster with trapped EVs was allowed to sediment for 15 s to get closer to the exit of the capillary, and then eluted in 100 μL of PBS at 5000 μL/min. The total processing time in the acoustic trap was ∼12.5 min per sample. The EVs were then analyzed using mass spectrometry.

### Transmission Electron Microscopy

EVs isolated from plasma from healthy mice using the acoustic trapping platform were imaged using transmission electron microscopy (TEM, FEI Tecnai Biotwin 120 kV). Copper TEM grids were treated with piliform, carbon-coated and glow-discharged. Samples were fixed with 4% PFA in a 1:1 ratio for 10 min at room temperature. Each grid was floated on top of 10 μL sample for 20 min. The grids were then floated on drops of PBS (5 min × 3), followed by fixation in 1% glutaraldehyde (5 min), deionized water (5 min) and stained in 1% uranyl acetate (5 min). The samples were imaged once dry.

### Nanoparticle Tracking Analysis

Nanoparticle tracking analysis (NTA, LM10, Malvern Analytical) was used to provide a size distribution of particles isolated in the acoustic trap. Trapped plasma samples from healthy mice were measured at camera level 15. Three 90 s videos were recorded and analyzed with detection threshold 4.

### Sample Preparation for Mass Spectrometry

Original plasma samples were diluted by adding 98 μL PBS to 2 μL plasma. The EVs in the trapped samples, as well as the original plasma samples, were lysed by adding 200 μL of RIPA buffer (Sigma-Aldrich) for 10 min followed by mechanical disruption in a Bioruptor Plus (Diagenode) for 20 cycles (30 s on, 30 s off) using the high setting. 1200 μL of −20 °C acetone was added to each sample before incubation at −20 °C overnight. The samples were then centrifuged at 18,200*g* for 30 min at 4 °C. The supernatant was removed and 500 μL of −20 °C 99.5% ethanol was added to the pellet, and the samples were centrifuged again at 18,200*g* for 30 min at 4 °C. The supernatant was removed, and the samples were dried in a SpeedVac (miVAC DUO) at 40 °C for 10 min and resuspended in 100 μL PBS.

The EV and corresponding plasma samples were prepared for mass spectrometry analysis by proteolytically digesting the proteins using trypsin double digestion. In total, 4.6 μL of solution containing 50 mM ammonium bicarbonate (ABC) and 10 M urea in LC-grade water, along with 2 μL of 0.5 μg/μL sequencing grade trypsin (Promega) were added to the samples and incubated for 30 min at 37 °C. Then, 45.4 μL of the ABC-urea solution were added to the samples and incubated for 30 min at room temperature. Next, the cysteine bonds were reduced by addition of 0.5 μL 500 mM tris­(2-carboxyethyl)­phosphine (TCEP), giving a final concentration of 1.64 mM TCEP, and the samples incubated for 60 min at 37 °C. 1 μL of 500 mM iodoacetamide was added, giving a final concentration of 3.26 mM iodoacetamide. The samples were incubated for 30 min at room temperature in the dark. The samples were diluted with 250 μL of 100 mM ABC and 2 μL of trypsin were added prior to incubation for 16 h at 37 °C. The samples were acidified with 10% trifluoroacetic acid to a pH of 2–3 and the peptides were purified using C18 reversed phase columns (Harvard apparatus Ultra-Micro SpinColumns Silica C18). The purified peptides were dried in a SpeedVac and reconstituted in 10 μL of 2% acetonitrile, 0.2% formic acid. The peptide concentration was then measured using a spectrophotometer (DeNovix, DS-11 FX+) to ensure injection of an equal amount of peptide (0.5 μg) into the mass spectrometer.

### Liquid Chromatography Tandem Mass Spectrometry (LC-MS/MS)

The peptides from each sample were analyzed on a Q Exactive HFX (Thermo Scientific) connected to an UltiMate 3000 RSLCnano System (Thermo Scientific) using data-independent mass spectrometry analysis (DIA-MS). Peptides were separated on a Thermo EASY-Spray column (Thermo scientific 50 cm column) at 45 °C and maximum pressure of 800 bar. A linear gradient of 4% to 45% acetonitrile in 0.1% formic acid was run for 50 min. A full MS scan of resolution 60,000 for a mass range of 390–1210 *m*/*z* was followed by 32 full fragmentation MS/MS scans (resolution 30,000) with a 26 *m*/*z* isolation window, including a 0.5 *m*/*z* overlap between windows. The precursor ions in the isolation windows were fragmented using higher-energy collision-induced dissociation with normalized collision energy of 30. Automatic gain control was set to 3e6 for MS and 1e6 for MS/MS.

### Mass Spectrometry Data Analysis

Data were stored and managed using openBIS.^28^ DIA-NN 1.8.1 ^29^ was used for identification and quantification, using a predicted spectral library from the reference proteomes of , and serotype M1 (UniProt proteome IDs UP000000589_10090, UP00000195, UP000000750_301447), resulting in a library containing 28807 proteins. Trypsin was used to digest in silico, with cysteine carbamidomethylation set as a fixed modification and allowing 1 missed cleavage. Minimum and maximum peptide length was 7 and 30, respectively. Mass tolerance for spectral matching was determined by DIA-NN 1.8.1 and set to <6.4 ppm for all samples. FDR was estimated by calculating *Q*-values comparing target and decoy peaks. The data was filtered at 1% false discovery rate (FDR) at precursor and protein level. The retention time (RT) was calibrated using iRT peptides. Nonproteotypic peptides were discarded. Protein intensities were quantified from the DIA-NN report with DPKS^30^ using the MaxLFQ method for protein level quantification. The resulting protein intensity matrix was then exported and analyzed using RStudio 4.3.2. In total, 861 proteins were identified across all samples. Proteins identified with only 1 peptide were discarded, leaving 641 proteins for further analysis. No minimum intensity threshold was set, and missing values were assigned an intensity of 0. The samples were analyzed, comparing differences between trapped samples versus plasma samples, infected samples versus healthy samples, and finally Gram-positive (GAS) versus Gram-negative () samples.

## Results

### Pathophysiological Response to Systemic Bacterial Infection

Invasive infection and sepsis are heterogeneous conditions, therefore, we used two mouse models of sepsis to investigate the EV proteome after isolation by acoustic trapping. Mice were infected systemically by intraperitoneal injection with (Group A streptococcus, GAS) or and sacrificed at 18 h postinfection (h.p.i.) (Figure 1A). PBS was administered to the healthy control group. To characterize systemic bacterial infection, pathophysiological read-outs such as body weight, bacterial dissemination, blood counts, immune cell activation, proinflammatory cytokine levels, and organ damage markers were examined (Figure 1B–G).

**Figure 1:**
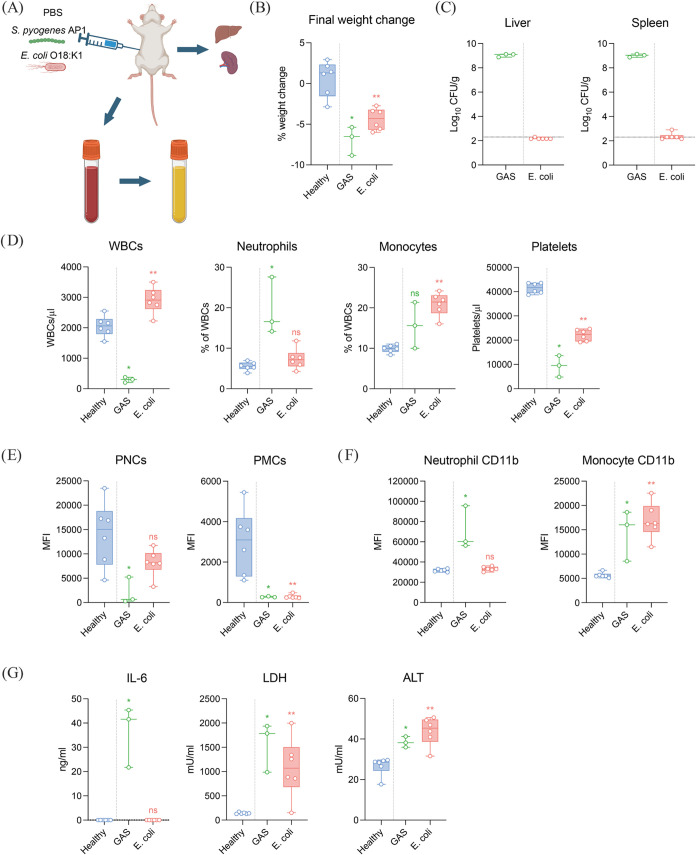
Pathophysiological response to systemic bacterial infection. (A) Nine-week-old female C57BL/6J mice were infected with 1 × 10^7^ CFU of AP1 (Group A streptococci, GAS; *n* = 3) or 1 × 10^4^ CFU of O18:K1 (*n* = 6) by intraperitoneal injection. Control mice were administered with PBS (*n* = 6). Animals were sacrificed at 18 h postinfection (h.p.i.). Blood was taken by cardiac puncture into citrate tubes and plasma was prepared from citrated blood. Liver and spleen were collected for homogenization. Figure created with Biorender.com. (B) Weight change was calculated as the final body weight at 18 h.p.i. subtracted from the initial weight before infection. (C) Bacterial load in the spleen and liver was determined by viable plate counts of organ homogenates. Dotted line represents the limit of detection. Flow cytometry was used to determine (D) total leukocyte (white blood cell, WBC) count per μL of sample, % circulating neutrophils and monocytes of total leukocyte counts, and platelet counts per μL of sample; (E) the median fluorescence intensity (MFI) of the formation of platelet-neutrophil complex (PNCs) and platelet-monocyte complexes (PMCs); and (F) the MFI of CD11b on neutrophils and monocytes. (G) Plasma was prepared from citrated blood and levels of IL-6, LDH activity, and ALT activity were determined using commercial kits. Data are shown as the median ± interquartile range. Statistical analysis was performed using Mann–Whitney test; ns = not significant, * *P* < 0.05, ** *P* < 0.01.

Body weight loss is a marker of overall health status during murine systemic infection.^31^ Body weight was determined prior to infection and 18 h.p.i. and the percentage of weight loss over time was calculated (Figure 1B). As expected, there was significant weight loss in animals infected with bacteria as compared to healthy animals, demonstrating a decline in the health status in the infected animals. To assess bacterial dissemination from the local peritoneal site of administration, bacterial load in distant organs (liver and spleen) was determined by viable plate counts (Figure 1C). Bacteria were present at elevated levels in the liver and spleen of animals infected with GAS, whereas animals infected with had levels of bacteria that were at or below the detection limit.

Flow cytometry was used to determine blood counts (Figure 1D) and immune cell activation (Figure 1E,F). Total leukocytes were identified according to characteristic size (FSC-A) and granularity (SSC-A). Neutrophils were identified as Ly-6G and Ly-6C high. Monocytes were identified within a lymphocyte/monocyte population as CD11b high. Platelets were identified as CD41-positive events. GAS infection led to a significant decrease in white blood cell (WBC) counts (leukopenia) and an increase in the percentage of neutrophils (neutrophilia) and monocytes (monocytosis) of WBCs as compared to healthy animals. A significant decrease in platelet counts (thrombocytopenia) was observed. Activated platelets can bind to circulating leukocytes, leading to the formation of platelet-neutrophil complexes (PNCs) and platelet-monocyte complexes (PMCs). PNCs and PMCs were identified as CD41-positive events on neutrophils and monocytes. PNC formation has previously been shown increase during early stages of invasive GAS infection and decrease below healthy baseline levels during late-stage infection^26^ similar to the significant decrease in PNCs and PMCs observed in the present study. There was a significant upregulation of CD11b on the surface of neutrophils and monocytes, indicating activation of these immune cells. Collectively, these data are in agreement with late-stage systemic infection with GAS.^26^ infection led to changes associated with early stage systemic infection including significant leukocytosis (increased WBC counts), monocytosis, and thrombocytopenia but no changes in circulating neutrophils as compared to healthy animals. A significant decrease in PMCs and upregulation of CD11b on monocytes was observed as compared to healthy animals, whereas no significant differences were observed for PNCs and neutrophil CD11b, suggesting an engagement of monocytes but not neutrophils in the infection model. This is likely due to the low levels of circulating neutrophils at this early stage.

Plasma was analyzed by ELISA for IL-6, an important mediator of the acute phase response to infection, and by colorimetric assay for lactate dehydrogenase (LDH) activity and alanine transaminase activity (ALT), markers for organ damage (Figure 1G). Animals infected with GAS had a significant increase in plasma levels of IL-6 and organ damage markers as compared to healthy animals, whereas animals infected with had a significant increase in organ damage markers but not IL-6, indicating organ damage in the absence of excess cytokine mobilization.

Altogether, these data demonstrate that GAS led to severe systemic infection in line with previous reports of murine streptococcal sepsis,^26,32^ whereas led to a milder systemic infection at the 18 h.p.i. end point in our models.

### Comparative Proteome Profiling of Acoustically Trapped Samples and Plasma Samples

In the next step, we asked whether the systemic infection resulted in changed protein content of EVs, and if acoustically trapped EVs could reveal additional information than the harvested plasma samples.

First, particle analysis of the trapped samples was performed using nanotracking analysis (NTA) and transmission electron microscopy (TEM), Figure 2. NTA showed a peak in the particle size distribution at 72 nm, which is in the expected size range for EVs. TEM revealed intact EVs isolated by the acoustic trapping platform. Heatmaps detailing the depletion of lipoproteins in trapped samples, as well as detection of the top 100 exosomal proteins from Exocarta can be found in Figures S1 and S2.

**Figure 2:**
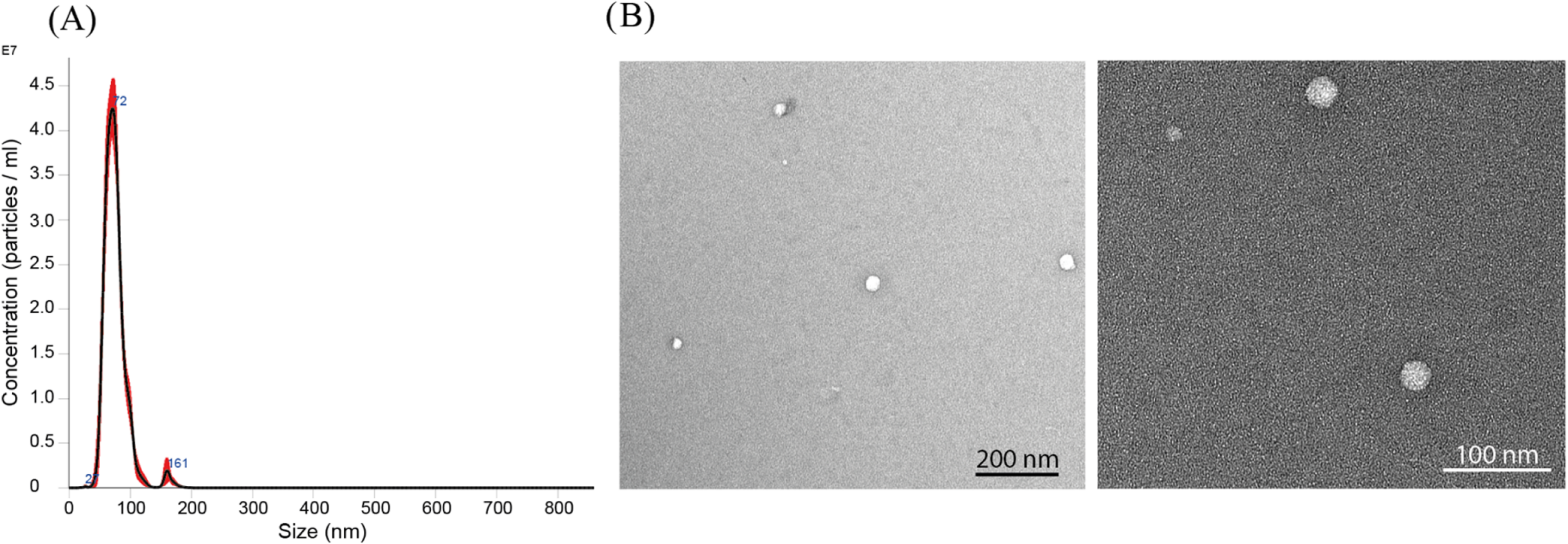
Particle analysis of trapped samples from healthy mouse plasma. (A) Size distribution of particles as measured by nanoparticle tracking analysis (NTA). The peak of the size distribution is 72 nm, which falls in the size range expected from EVs. (B) Transmission electron microscopy (TEM) images showing EVs isolated by the acoustic trapping platform.

An overview of the sample processing can be seen in Figure 3A. EVs were isolated from background plasma using the acoustic trapping platform. The trapped samples and the corresponding untrapped plasma samples (referred to as plasma samples) were then analyzed using quantitative data independent acquisition mass spectrometry (DIA-MS). A principal component analysis (PCA) of all samples shows that the largest differences were between trapped samples and plasma samples, indicating that acoustic trapping enriches for distinct proteomes (Figure 3B). The plasma samples further cluster together depending on condition (GAS, , or Healthy), indicating clear differences in protein content between these groups.

**Figure 3:**
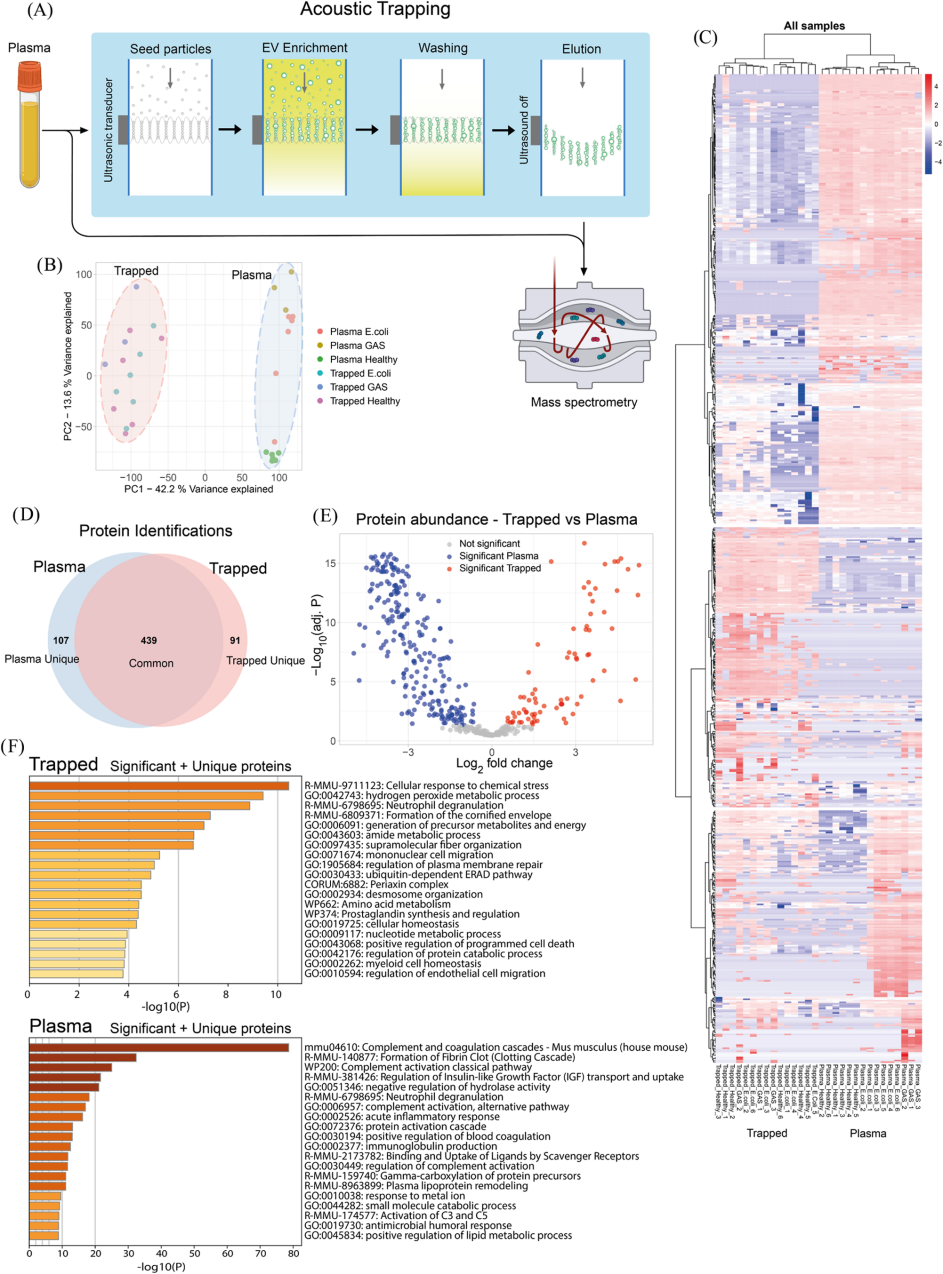
Comparison of trapped samples and the untrapped plasma samples. (A) Schematic overview of plasma sample processing in the acoustic trap. Vesicles are trapped in the ultrasonic standing wave to facilitate enrichment and washing. The protein content of the trapped samples and the untrapped plasma samples was then analyzed with mass spectrometry. (B) Principal component analysis of all samples. (C) Heatmap of all protein intensities across all samples with clustered using Ward’s method (ward.d2 in R). Intensities have been log 2 transformed and row normalized. Missing values have been assigned an intensity of 0 and the legend gives the *z*-score. (D) Venn diagram of identified proteins across all trapped samples and all plasma samples. Proteins were counted as present within a group if the protein was identified in at least 2 samples within the group. (E) Volcano plot comparing all proteins found in common between trapped samples and plasma samples. The p-value has been adjusted for multiple hypothesis testing (Benjamini-Hochberg). Differentially abundant proteins were determined to be significant if the adjusted p-value was less than 0.05. (F) Functional enrichment analysis of all proteins that were significantly abundant between trapped samples and plasma samples along with all proteins uniquely expressed in the respective group. Keratin proteins have been excluded from the analysis.

A protein intensity heatmap for all samples can be found in Figure 3C. A larger version of the heatmap, where individual proteins can be seen, is provided in Figure S4. The heatmap is row normalized and the legend gives the z-score after assigning missing values an intensity of 0. The samples and proteins have been clustered using unsupervised clustering. The samples cluster in accordance with the pattern in the PCA plot in Figure 3B, with clear distinction between trapped samples and plasma samples, and a clear clustering within the plasma samples depending on condition. The heatmap reveals clusters of proteins with a clear difference between trapped samples and plasma samples, showing that proteins are enriched or washed away in the trap, giving access to different compartments of the proteome.

In total, 107 proteins were uniquely found across all plasma samples, and 91 proteins were uniquely found across all trapped samples when including proteins that were identified in at least 2 samples within a group (Figure 3D). Differential abundance analysis between the trapped samples and plasma samples revealed 76 significant proteins that were enriched in the trapped samples (Figure 3E). The *p*-value has been adjusted for multiple hypothesis testing (Benjamini-Hochberg) and proteins were determined to be significantly abundant if the adjusted p-value was lower than 0.05. Functional enrichment analysis^33^ of all proteins in the trapped samples that were significantly abundant (Figure 3E), along with uniquely identified (Figure 3D), demonstrates that the trapped samples contain mainly proteins related to cellular response to chemical stress, cellular homeostasis, plasma membrane repair and mononuclear cell migration (Figure 3F) indicative of cellular proteins and processes. Keratin proteins have been excluded from analysis, because of the high likelihood that they originate from contaminants. In contrast, the plasma samples contain mainly proteins related to complement and coagulation cascades, regulation of insulin-like growth factor, neutrophil degranulation, and acute inflammatory response, which are known to be present at high amounts in the circulation.

In summary, these data show that the isolation of EVs with the acoustic trap provides information and access to more cellular proteins from a plasma sample, which were not obtained from the plasma samples alone.

### Comparative Proteome Profiling of Infected and Healthy Samples

After verifying that acoustically trapping the plasma samples gave access to a different proteome, we investigated these differences for samples taken from mice with systemic infection (infected samples) versus samples taken from mice injected with PBS (healthy samples) to quantify how systemic infection or sepsis alters the proteome in both plasma and EVs.

Principal component analyses of trapped samples and plasma samples demonstrate that the proteomics data cluster based on condition (Healthy, GAS, ) for both sample types (Figure 4A) when performing the analysis individually for trapped and plasma samples. Heatmaps of all protein intensities for trapped samples and plasma samples can be found in Figure S5. For trapped samples, 36 proteins were found uniquely in healthy samples, and 74 proteins were found uniquely within infected samples (GAS, ). For plasma samples, 6 proteins were found uniquely in healthy samples, and 141 proteins were found uniquely within infected samples (Figure 4B). In this analysis, a protein was determined to be present within a group if it was identified within at least 2 samples in the group.

**Figure 4:**
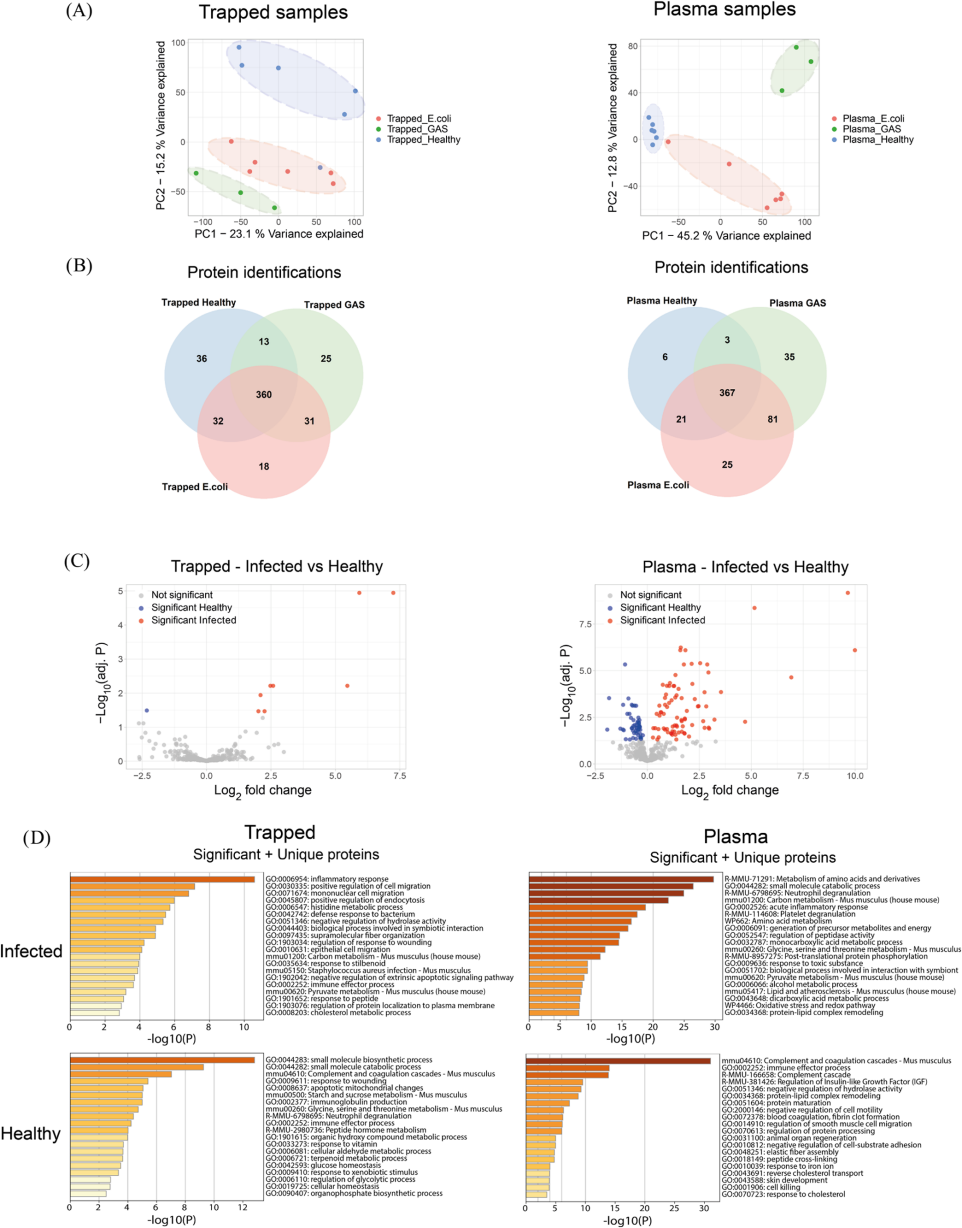
Comparison of infected samples and healthy controls within both trapped samples and plasma samples. (A) Principal component analysis of all trapped samples and all plasma samples, respectively. (B) Venn diagram comparing protein identifications within trapped samples and plasma samples. A protein was determined to be present in a group if identified in at least 2 samples within the group. (C) Volcano plots comparing protein abundances in samples from healthy mice and infected mice, for trapped and plasma samples, respectively. The p-value has been adjusted for multiple hypothesis testing (Benjamini-Hochberg). Differentially abundant proteins were determined to be significant if the adjusted p-value was less than 0.05. (D) Functional enrichment analyses of all proteins that are significantly abundant and uniquely found within the respective group (Trapped Infected vs Trapped Healthy and Plasma Infected vs Plasma Healthy).

Differential protein abundance analysis comparing infected and healthy samples for both trapped and plasma samples showed that systemic infection introduced marked changes in both the trapped and the plasma samples (Figure 4C). The p-values were adjusted for multiple hypothesis testing (Benjamini-Hochberg) and differentially abundant proteins were determined to be significant if the adjusted p-value was lower than 0.05. Functional enrichment analysis of all trapped infected samples that were significantly abundant within a group along with uniquely identified demonstrates that the trapped infected samples contained mainly proteins related to inflammatory response, mononuclear cell migration, endocytosis, and metabolism of histidine (Figure 4D). In contrast, plasma infected samples contained mainly proteins related to amino acid metabolism, neutrophil degranulation, and platelet degranulation. The trapped healthy samples contained mainly proteins related to small molecule biosynthetic processes, complement and coagulation, and starch and sucrose metabolism, whereas the healthy plasma samples were enriched for proteins related to complement and coagulation, regulation of insulin-like growth factors, and negative regulation of cell motility. This shows that systemic infection alters the proteomes in plasma and EVs differently.

In summary, these results show that systemic infection alters the proteome in both plasma and EVs, with a larger effect on the plasma protein content. In our models, systemic infection affected mainly metabolism, neutrophil degranulation, and inflammation in plasma samples, and mainly cell migration, endocytosis, and inflammation in trapped samples.

### Comparative Proteome Profiling of Gram-Negative and Gram-Positive Systemic Infection

In the final analysis, we investigated if the EV and plasma proteome are altered in a pathogen-specific manner. In this analysis, the proteins that were significantly abundant along with proteins uniquely expressed within a group (as seen in the Venn diagrams in Figure 4B) were subjected to functional enrichment analysis comparing samples from mice infected with Gram-positive (GAS) and Gram-negative () bacteria (Figure 5A). Specifically, the trapped GAS samples were compared to trapped samples, and the plasma GAS samples were compared to plasma samples. Samples from GAS-infected mice with severe systemic infection contained mainly proteins related to inflammatory response and mononuclear cell migration for both trapped samples and plasma samples. Trapped samples from -infected mice with mild systemic infection showed mainly proteins related to complement and coagulation cascades, glucose homeostasis, and retinoid metabolic processes. Plasma samples from infected mice contained mainly proteins related to complement and coagulation cascades and fatty acid metabolism.

**Figure 5:**
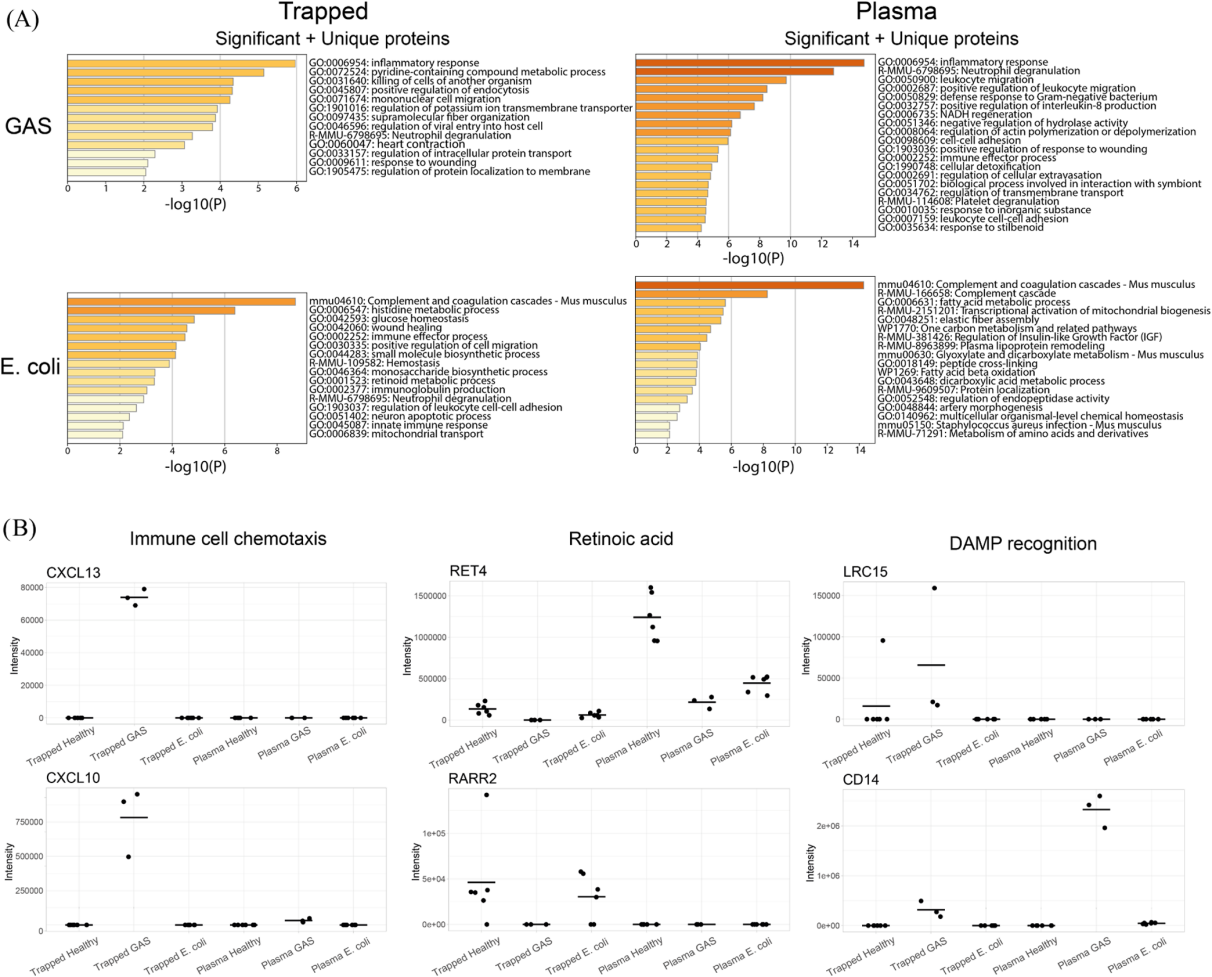
Comparison of Gram-positive (GAS) and Gram-negative () samples. (A) Metascape analyses of proteins significantly enriched when comparing samples from mice infected with GAS with mice infected with . Trapped samples have been compared against trapped samples, and plasma samples have been compared against plasma samples. Proteins with an adjusted p-value <0.05 along with proteins uniquely expressed within each group has been included in the analysis. (B) Protein intensities of selected proteins related to immune cell chemotaxis, retinoic acid, and DAMP recognition.

To further investigate specific enriched ontological terms found in the functional enrichment analysis, the data set was filtered on mononuclear cell migration and retinoid metabolic process, and displayed in heatmaps, Figures S6 and S7. Intensities of selected individual proteins found within these heatmaps highlight specific biological functions as seen in Figure 5B. Chemokine CXCL13 was exclusively detected in trapped GAS samples, and CXCL10 was exclusively found in GAS samples, with greater intensity in trapped GAS samples compared to plasma GAS samples. Retinoic acid related proteins RET4 (retinol binding protein) and RARR2 (retinoic acid responder protein) were both depleted in trapped GAS samples, but present in other trapped samples. LRC15 (leucine-rich repeat containing protein) was abundant in trapped GAS samples but no other groups, and CD14 (monocyte differentiation antigen) was abundant in all GAS samples but no other groups. Both LRC15 and CD14 contribute to recognition of damage associated molecular pathways (DAMPs). These results show that the Gram-positive GAS infection model used in this study caused a stronger inflammatory response and neutrophil activation, while the Gram-negative infection model had stronger effects on metabolism.

## Discussion

In this study, we demonstrate that acoustic trapping can isolate EVs from murine plasma to reveal an additional repertoire of proteins that are regulated during systemic bacterial infection from a single blood sample. There were clear differences in the protein content in samples that were processed in the acoustic trap as compared with the original plasma sample. This can be seen in Figure 3, with large groups of proteins that are enriched or depleted in the acoustic trap. Additionally, 91 proteins were uniquely found within the trapped samples, which shows that trapping the samples provides deeper access to the proteome than plasma samples alone. This can be explained by the enrichment of EVs and removal through washing of abundant plasma proteins that obscure quantification of proteins found within the EV population. Functional enrichment analyses demonstrate that the EV-enriched proteome is comprised of cellular-derived proteins. EVs contain proteins from their cell of origin which provides new opportunities to probe proteome changes reflecting the cellular state directly in samples that can be collected in the clinical routine. Here, functional enrichment demonstrates that disparate cellular processes such as response to chemical stress, regulation of plasma membrane repair, prostaglandin synthesis and regulation and cellular homeostasis can be quantified in the EV proteome. These terms were not enriched in the original untrapped plasma proteomes, further augmenting the additional proteome responses that can be uncovered in the enriched EV populations. The enriched terms are also different than in previous studies on acoustically isolated platelet derived EVs,^24^ demonstrating the value of isolating a broad EV population when studying complex conditions, such as sepsis. It could be emphasized that mass spectrometry based proteomics can be performed on very small volumes of plasma (1 μL), and therefore it should be possible to run analyses on both the EV fraction and the original plasma sample in parallel, even when the available sample volume is low. The result would then simply be that more information can be recovered from a plasma sample.

Bacterial infection introduced a significant effect on the inflammatory response and metabolism on the protein level. Severe systemic GAS infection generated a robust immune response in our models, which was seen through enrichment of proteins associated with the inflammatory response and neutrophil degranulation. This agrees well with the pathophysiological read-outs (Figure 1) indicating that GAS-infected mice had severe systemic infection. In contrast, led to a milder infection in the mice. On the protein level, stronger effects on metabolism were observed in -infected animals, which was seen through enrichment of proteins related to metabolism of fatty acids and amino acids. This is in line with Gram-negative bacteria releasing lipopolysaccharide (LPS) that have been shown to affect metabolism and promote lipolysis.^34,35^ The effect on metabolism was not as pronounced in GAS-infected mice, possibly due to the fact that Gram-positive bacteria do not release LPS.

Systemic bacterial infection altered the proteomes of EVs and plasma differently (Figure 5D). Comparing infected to healthy, the significant and unique proteins were mainly related to metabolism of amino acids, acute inflammatory response and neutrophil and platelet degranulation. In contrast, systemic infection in trapped samples altered mainly proteins related to regulation of cell migration, endocytosis, defense response to bacterium and inflammation. This again demonstrates that the acoustic trapping platform enables access to information that is normally not obtainable from a plasma sample, and indicates which biological functions EVs play a role in during systemic bacterial infection.

Immune cell signaling and recruitment is, to some extent, mediated through EVs. This can be seen in the functional enrichment analyses, revealing proteins related to mononuclear cell migration for all trapped samples. Additionally, CXCL13 and CXCL10, both strong chemokines, were found almost exclusively in trapped GAS samples. Interestingly, this effect is not seen when only considering plasma samples in our model, highlighting the importance of considering biological processes at the EV level. CXCL10 has previously been reported to be found in serum samples from septic mice in an LPS challenge model.^36^

Retinoic acid related proteins, which affect macrophages and regulation of inflammation, are depleted in EVs from trapped GAS samples. Retinoic acid signaling in leukocytes has previously been shown to be altered during sepsis.^3^ Using the acoustic trap, this effect can be seen directly from plasma EVs. Additionally, DAMP recognition proteins are present in EVs from trapped GAS samples. The depletion of proteins that regulate inflammation from EVs, along with the presence of DAMP-recognizing proteins, may contribute to the dysregulated and systemic inflammation observed in sepsis.

In this study we showed that new information is uncovered when analyzing the EVs present in plasma during sepsis, and that the changes in protein signal in EVs and plasma are different from one another. Additionally, it shows that the acoustic trapping method provides easy access to the plasma EV compartment and therefore makes it feasible to study EVs changes in EVs during sepsis, especially when a high number of samples need to be processed and the available sample volume is low. However, the study should be seen as a proof-of-concept study and consequently, the observations made here need to be confirmed in additional future studies.

National efforts have been launched to coordinate the collection and annotation of clinical samples to deepen the understanding of the variability in the sepsis disease progression.^37^ These activities have further catalyzed population-scale proteomics studies that, along with recent developments in explainable artificial intelligence, enables the identification of hidden molecular patterns in the blood plasma that can be used to predict disease outcome at the time of hospital admission.^30^ The addition of data from EVs in sepsis patients could provide valuable insights about disease progression. Acoustic trapping provides easy access to EVs from biobanked samples, enabling studies on the large cohorts of plasma samples collected on a national level. Future studies optimizing the mass spectrometry sample preparation coupled with acoustically trapped samples could decrease processing time and further facilitate large scale proteomic profiling of EVs in sepsis patients.

## Conclusions

In this study, we showcase that analyzing EVs present in plasma reveals information about biological processes that cannot be observed when analyzing plasma alone. The small sample volume of 80 μL required in the acoustic trap enables EVs to be isolated from biobanked clinical sepsis samples. Getting access to more information from a plasma sample could aid in understanding the complex mechanisms that take place in an organism during sepsis. The potential use of EV proteomes for improving diagnostics and treatment of patients suffering from sepsis needs, however, to be further explored in clinical samples from sepsis patients.

## Supplementary Material



## Data Availability

Raw MS files along with DIA-NN search results, the spectral library, the FASTA file, protein identification and intensity tables are deposited in MassIVE database (doi:10.25345/C5D795N90).

## Notes

Protein intensity heatmap of all identified proteins related to lipoproteins (Figure S1); protein intensity heatmap of all identified proteins on ExoCarta (Figure S2); gating strategies for flow cytometry measurements (Figure S3); full size protein intensity heatmap of all samples (Figure S4); protein intensity heatmap of trapped samples and plasma samples respectively (Figure S5); protein intensity heatmap of all identified proteins related to mononuclear cell migration (Figure S6); protein intensity heatmap of all identified proteins related to retinoid metabolic process (Figure S7) (PDF)

## Abbreviations

ABC: ammonium bicarbonate
ALT: alanine transaminase
DAMP: damage-associated molecular pathway
EV: extracellular vesicle
LDH: lactate dehydrogenase
GAS: group A streptococcus
PMC: platelet-monocyte complex
PNC: platelet-neutrophil complex
SEC: size exclusion chromatography
TCEP: tris­(2-carboxyethyl)­phosphine
UC: ultracentrifugation
WBC: white blood cell
