# Supplementary material for: Proteomic Profiling of Acoustically Isolated Extracellular Vesicles from Blood Plasma during Murine Bacterial Sepsis
Source: J Proteome Res. 2025 Jul 11;24(8):4126–38. doi: 10.1021/acs.jproteome.5c00267 (PMC12322956; doi:10.1021/acs.jproteome.5c00267)
Supplement: Supplementary file 1 [file pr5c00267_si_001.pdf]

Supporting Information

Proteomic profiling of acoustically isolated extracellular vesicles from blood plasma during murine bacterial sepsis

Axel Broman[1*], Yashuan Chao[2,3], Oonagh Shannon[3], Thomas Laurell[1] and Johan Malmström[2*]

1. Department of Biomedical Engineering, Lund University, Lund 222 42, Sweden
2. Division of Infection Medicine, Department of Clinical Sciences, Lund University, Lund 222 42, Sweden
3. Section of Oral Biology, Faculty of Odontology, Malmö University, Malmö 205 06, Sweden

* *Corresponding author(s): axel.broman@bme.lth.se, johan.malmstrom@med.lu.se*

<u>Supporting Figures</u>

Figure S1: Protein intensity heatmap of all identified proteins related to lipoproteins.

Figure S2: Protein intensity heatmap of all identified proteins on ExoCarta.

Figure S3: Gating strategies for flow cytometry measurements (Related to Figure 1).

Figure S4: Full size protein intensity heatmap of all samples (Related to Figure 2).

Figure S5: Protein intensity heatmaps of trapped samples and plasma samples respectively (Related to Figure 3).

Figure S6: Protein intensity heatmap of all identified proteins related to mononuclear cell migration (Related to Figure 4).

Figure S7: Protein intensity heatmap of all identified proteins related to retinoid metabolic process (Related to Figure 4).

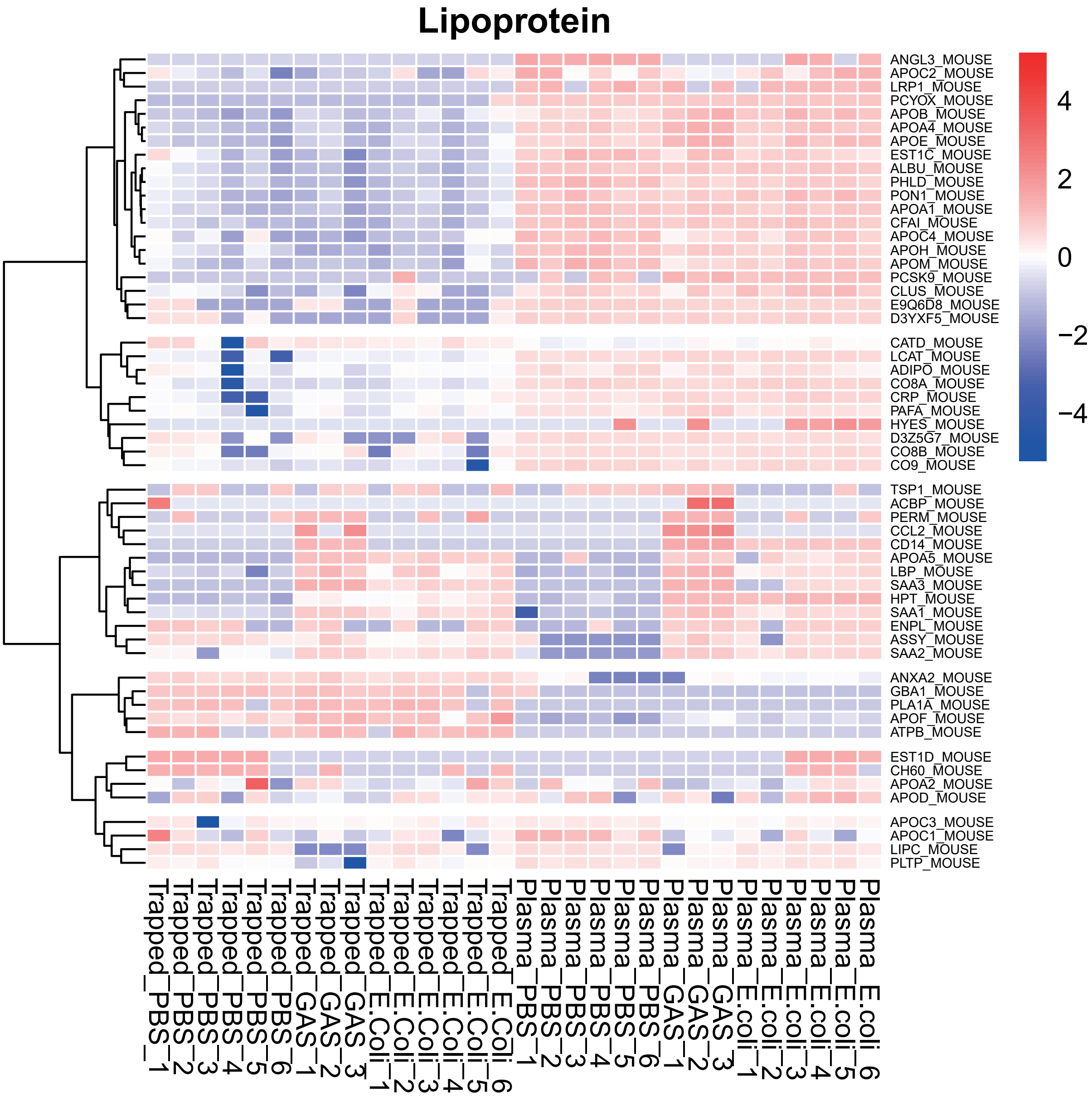

Figure S1: Heatmap of all detected proteins that are associated with lipoprotein function according to Mouse Genome Informatics (MGI) (https://www.informatics.jax.org/). The rows are clustered via unsupervised clustering and the columns are clustered manually. Intensities are log2 transformed and row normalized, and the legend gives the z-score. Missing values have been assigned an intensity of 0.

ExoCarta Top 100

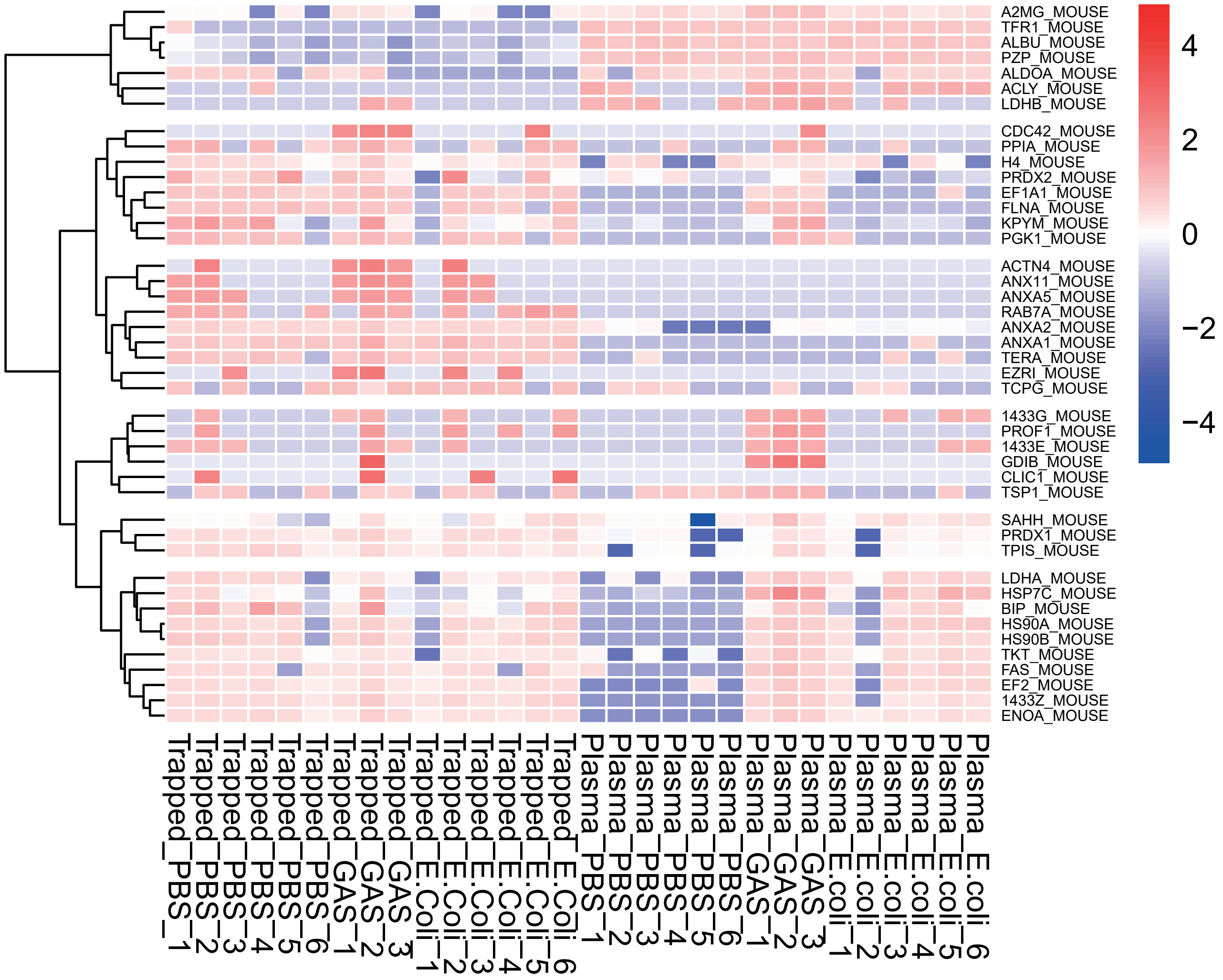

Figure S2: Heatmap of all detected proteins that are listed in the ExoCarta Top 100 proteins. The rows are clustered via unsupervised clustering and the columns are clustered manually. Intensities are log2 transformed and row normalized, and the legend gives the z-score. Missing values have been assigned an intensity of 0.

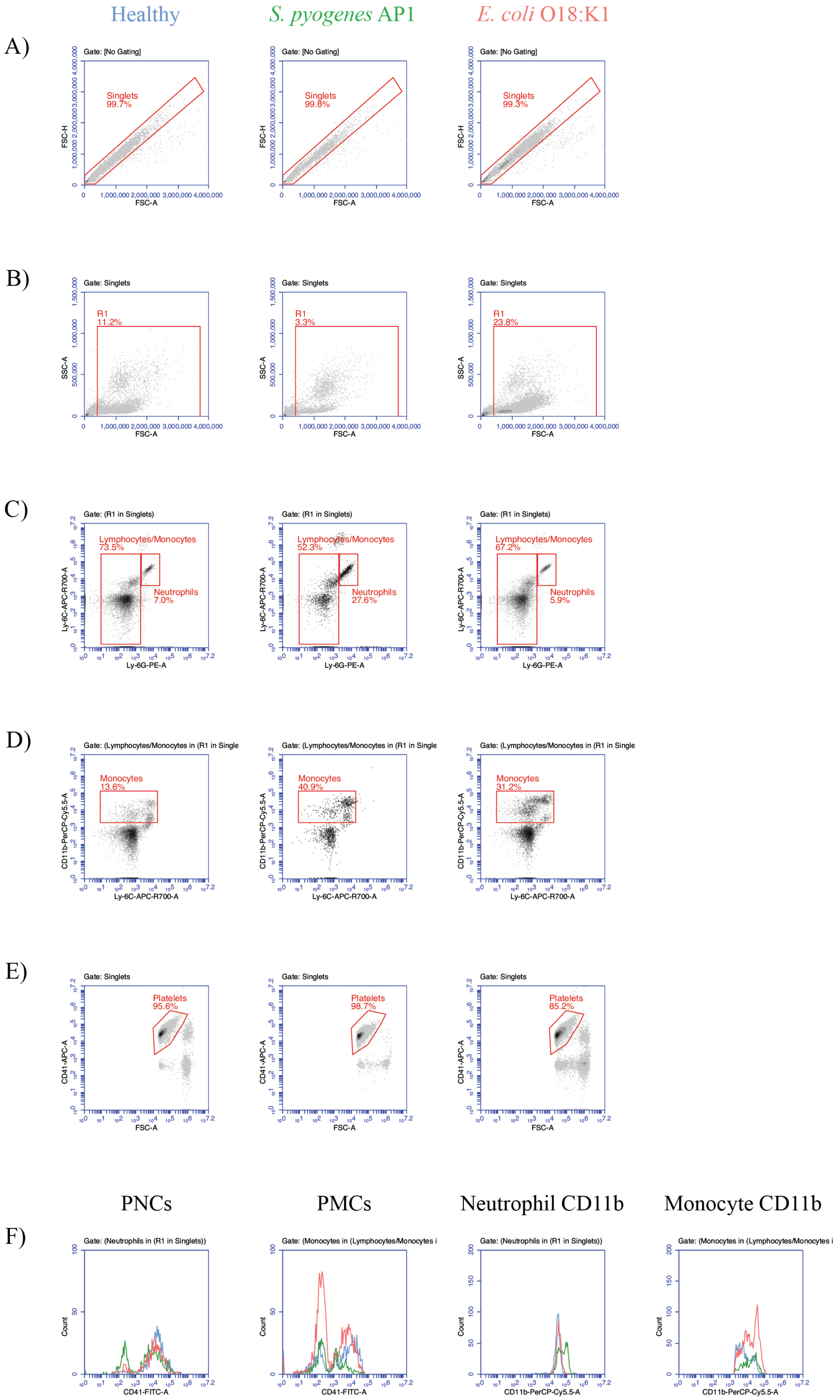

Figure S3: Representative gating strategies and histogram plots for whole blood. A) Singlets were gated using a FSC-A versus FSC-H plot. B) In the white blood cell panel, total leukocytes were gated (R1) according to characteristic FSC-A and SSC-A. C) From total singlet leukocytes (R1 in Singlets), neutrophils were gated as Ly-6G and Ly-6C high. Lymphocytes and monocytes were gated together as Ly-6G low and Ly-6C intermediate/high. D) Monocytes were distinguished from lymphocytes with CD11b. E) In the platelet panel, platelets were identified as FSC low and CD41 positive. F) The median fluorescence intensity (MFI) of CD11b (activation marker) and CD41 (platelet-positive events) in the neutrophil gate or monocyte gate was determined using histogram plots.

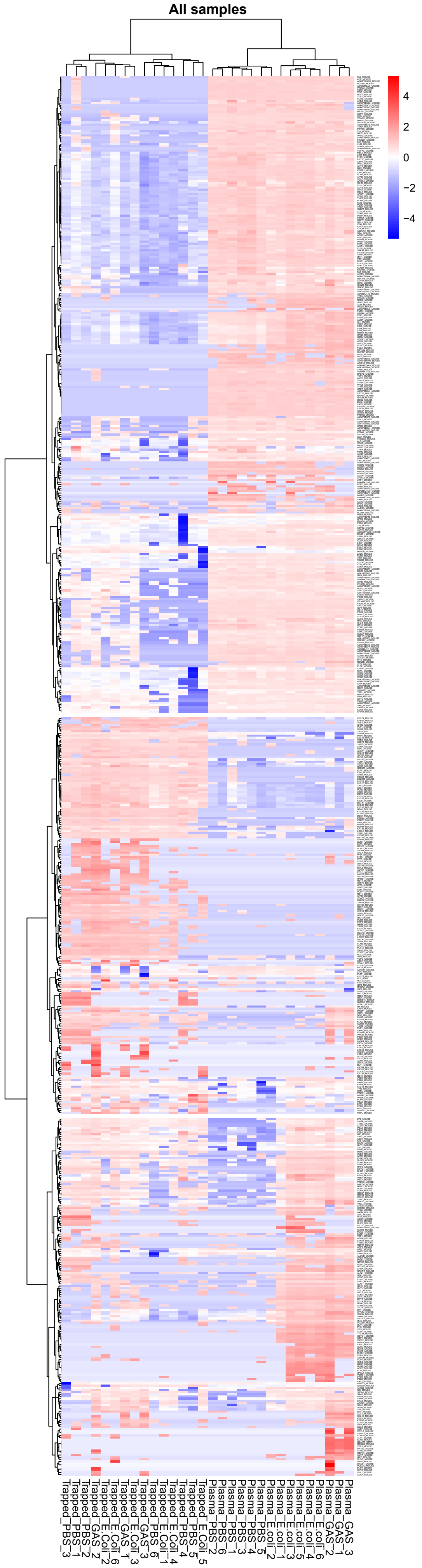

Figure S4: Protein intensity heatmap of all proteins detected in all samples, clustered via Ward's method (ward.d2 in R). Missing values have been assigned an intensity of 0. Intensities have been log2 transformed, the heatmap has been row normalized and the legend gives the z-score.

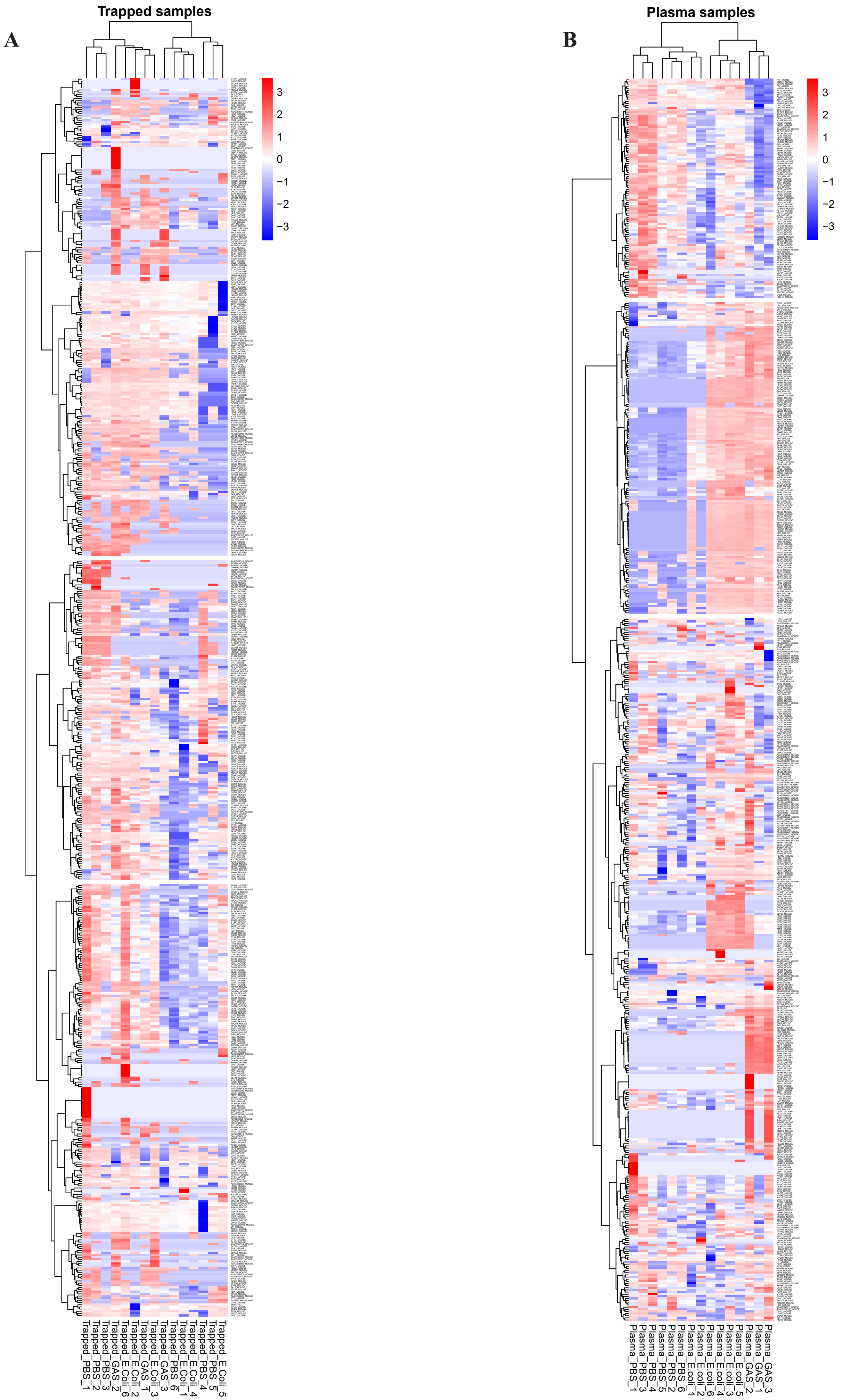

Figure S5: Protein intensity heatmaps of all proteins detected in (A) all samples processed on the acoustic trap, (B) all plasma samples. The heatmaps have been clustered via Ward's method (ward.d2 in R). Missing values have been assigned an intensity of 0. Intensities have been log2 transformed, the heatmaps are row normalized and the legend gives the z-score.

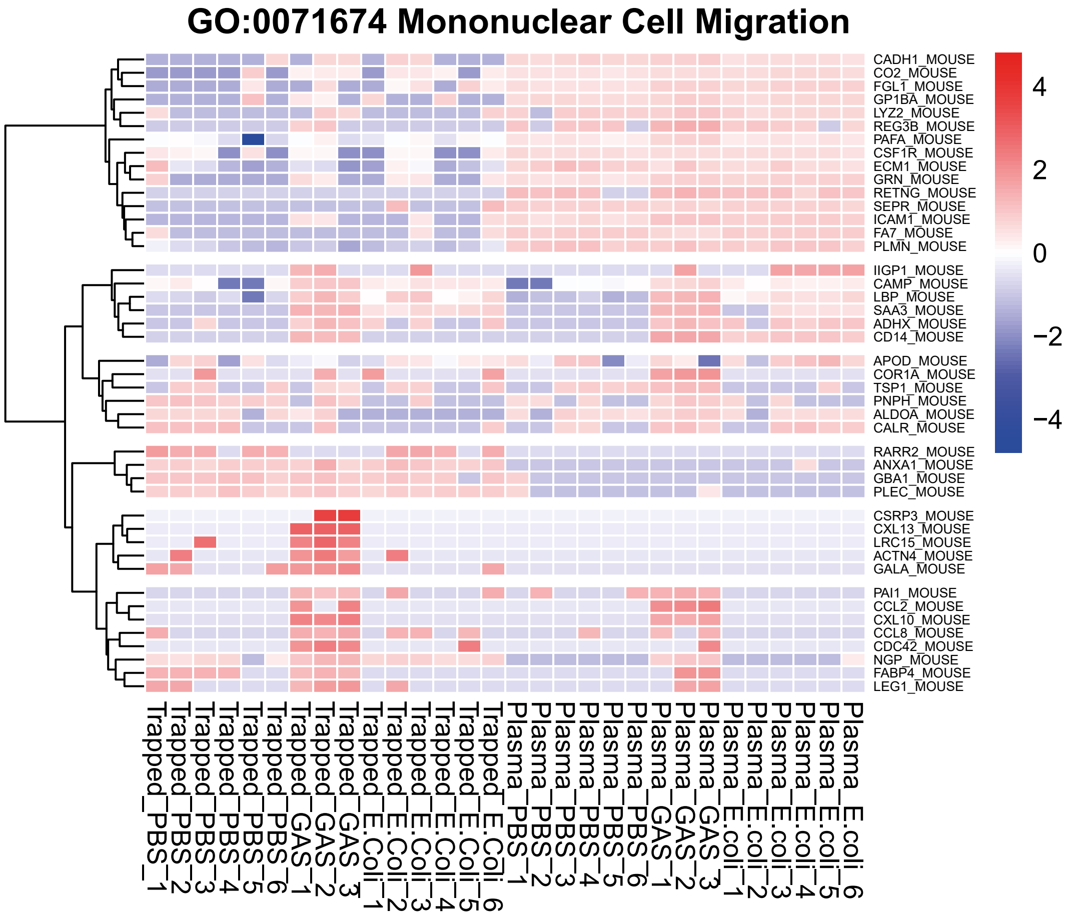

Figure S6: Protein intensity heatmap of all proteins detected related to mononuclear cell migration. The rows are clustered via Ward's method (ward.d2 in R) and the columns are clustered manually. Intensities are log2 transformed and row normalized, and the legend gives the z-score. Missing values have been assigned an intensity of 0.

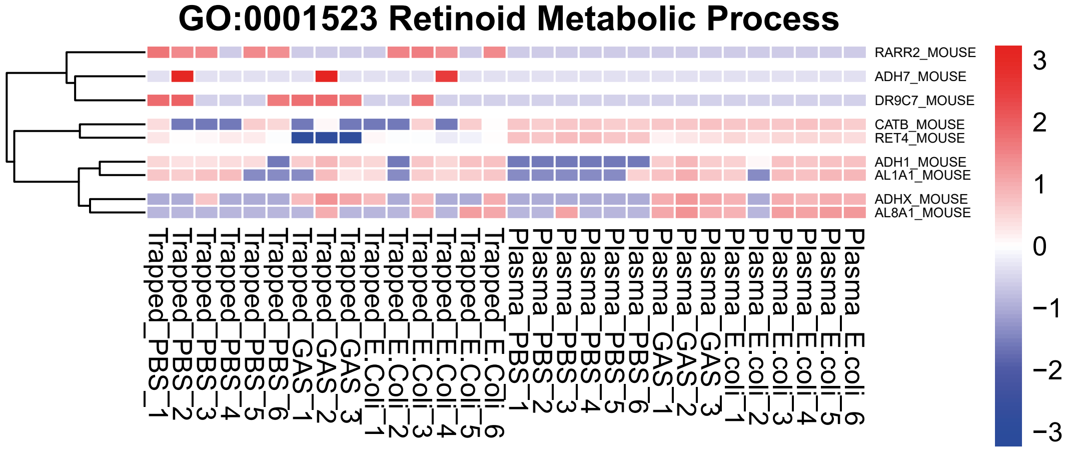

Figure S7: Protein intensity heatmap of all proteins detected related to retinoid metabolic processes. The rows are clustered via Ward's method (ward.d2 in R) and the columns are clustered manually. Intensities are log2 transformed and row normalized, and the legend gives the z-score. Missing values have been assigned an intensity of 0.
